# *N*-(4-Methyl­benzo­yl)-4-nitro­benzene­sulfonamide

**DOI:** 10.1107/S1600536812007854

**Published:** 2012-02-29

**Authors:** P. A. Suchetan, Sabine Foro, B. Thimme Gowda

**Affiliations:** a Department of Chemistry, Mangalore University, Mangalagangotri 574 199, Mangalore, India; b Institute of Materials Science, Darmstadt University of Technology, Petersenstrasse 23, D-64287 Darmstadt, Germany

## Abstract

In the title compound, C_14_H_12_N_2_O_5_S, the dihedral angle between the nitro­phenyl group and the –S—NH—C—O fragment is 80.74 (17)° and that between the nitro­phenyl and methyl­phenyl groups is 87.66 (14)°. The C—S—N—C torsion angle at the S—N bond is −67.0 (3)°. In the crystal, mol­ecules are linked into *C*(4) chains *via* N—H⋯O hydrogen bonds.

## Related literature

For our studies on the effects of substituents on the structures and other aspects of *N*-aryl­amides, see: Gowda *et al.* (1999 ▶, 2006 ▶). For *N*-aryl-methane­sulfonamides, see: Gowda *et al.* (2007 ▶). For *N*-(substituted-benzo­yl)-aryl­sulfonamides, see: Suchetan *et al.* (2010 ▶). For *N*-chloro­aryl­amides, see: Jyothi & Gowda (2004 ▶). For *N*-bromo­aryl­sulfonamides, see: Usha & Gowda (2006 ▶).
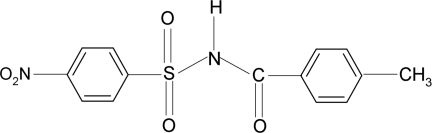


## Experimental

### 

#### Crystal data

C_14_H_12_N_2_O_5_S
*M* *_r_* = 320.32
Orthorhombic, 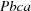

*a* = 13.969 (1) Å
*b* = 9.6591 (6) Å
*c* = 21.026 (2) Å
*V* = 2837.0 (4) Å^3^
*Z* = 8
Mo *K*α radiation
μ = 0.25 mm^−1^
*T* = 293 K
0.40 × 0.18 × 0.18 mm

#### Data collection

Oxford Diffraction Xcalibur diffractometer with a Sapphire CCD detector
Absorption correction: multi-scan (*CrysAlis RED*; Oxford Diffraction, 2009 ▶) *T*_min_ = 0.905, *T*_max_ = 0.956
7163 measured reflections
2864 independent reflections
1835 reflections with *I* > 2σ(*I*)
*R*_int_ = 0.027

#### Refinement

*R*[*F* ^2^ > 2σ(*F* ^2^)] = 0.062
*wR*(*F* ^2^) = 0.152
*S* = 1.16
2863 reflections
203 parameters
7 restraints
H atoms treated by a mixture of independent and constrained refinement
Δρ_max_ = 0.32 e Å^−3^
Δρ_min_ = −0.21 e Å^−3^

### 

Data collection: *CrysAlis CCD* (Oxford Diffraction, 2009 ▶); cell refinement: *CrysAlis RED* (Oxford Diffraction, 2009 ▶); data reduction: *CrysAlis RED*; program(s) used to solve structure: *SHELXS97* (Sheldrick, 2008 ▶); program(s) used to refine structure: *SHELXL97* (Sheldrick, 2008 ▶); molecular graphics: *PLATON* (Spek, 2009 ▶); software used to prepare material for publication: *SHELXL97*.

## Supplementary Material

Crystal structure: contains datablock(s) I, global. DOI: 10.1107/S1600536812007854/yk2044sup1.cif

Structure factors: contains datablock(s) I. DOI: 10.1107/S1600536812007854/yk2044Isup2.hkl

Supplementary material file. DOI: 10.1107/S1600536812007854/yk2044Isup3.cml

Additional supplementary materials:  crystallographic information; 3D view; checkCIF report

## Figures and Tables

**Table 1:** Hydrogen-bond geometry (Å, °)

*D*—H⋯*A*	*D*—H	H⋯*A*	*D*⋯*A*	*D*—H⋯*A*
N1—H1N⋯O3^i^	0.85 (2)	2.16 (2)	2.994 (4)	168 (4)

Symmetry code: (i) 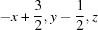.

**Fig. 1.**
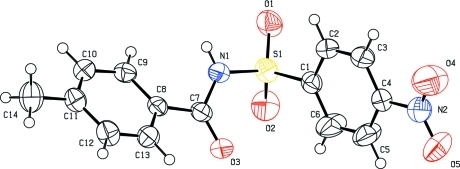
Molecular structure of the title compound, showing the atom- labelling scheme. Displacement ellipsoids are drawn at the 50% probability level.

**Fig. 2.**
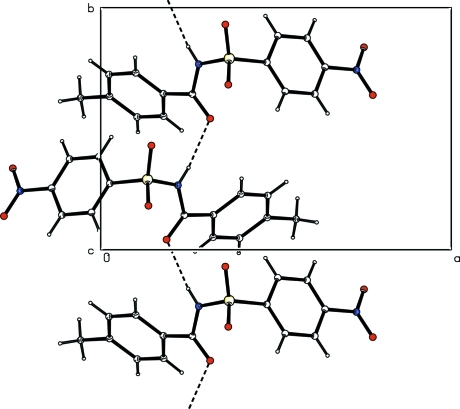
Molecular packing in the title compound. Hydrogen bonds are shown as dashed lines.

## supplementary crystallographic information

### Comment

As part of our studies on the substituent effects on the structures and other aspects of *N*-arylamides (Gowda *et al.*, 1999, 2006), *N*-aryl-methanesulfonamides (Gowda *et al.*, 2007), *N*-(substituted-benzoyl)-arylsulfonamides (Suchetan *et al.*, 2010), *N*-chloroarylsulfonamides (Jyothi & Gowda, 2004) and *N*-bromoarylsulfonamides (Usha & Gowda, 2006), in the present work, the crystal structure of *N*-(4-methylbenzoyl)-4-nitrobenzenesulfonamide has been determined (Fig.1).

The conformation of the N—H bond in the C—SO_2_—NH—C(O) segment is *anti* with respect to the C=O bond (Fig.1), similar to that observed in *N*-(4-methylbenzoyl)-4-chlorobenzenesulfonamide (I) (Suchetan *et al.*, 2010).

In the title compound, the molecules are twisted at the *S–N* bonds with the torsional angle of -67.0 (3)°, compared to the value of 69.0 (2)° in (I).

The dihedral angle between the sulfonyl benzene ring and the —SO_2_—NH—C—O segment is 79.6 (1)°, compared to the value of 77.2 (1)° in (I).

The dihedral angle between the sulfonyl and the benzoyl benzene rings is 89.3 (1)°, compared to the value of 89.5 (1)° in (I).

The packing of molecules linked by of N—H···O hydrogen bonds (Table 1) is shown in Fig. 2.

### Experimental

The title compound was prepared by refluxing a mixture of 4-methylbenzoic acid, 4-nitrobenzenesulfonamide and phosphorous oxy chloride for 3 h on a water bath. The resultant mixture was cooled and poured into ice cold water. The solid obtained was filtered, washed thoroughly with water and then dissolved in sodium bicarbonate solution. The compound was reprecipitated by acidifying the filtered solution with dilute HCl. It was filtered, dried and recrystallized.

Rod like colourless single crystals of the title compound used in X-ray diffraction studies were obtained by slow evaporation from its toluene solution at room temperature.

### Refinement

The H atom of the NH group was located in a difference map and restrained to N—H = 0.86 (2) Å. The other H atoms were positioned with idealized geometry using a riding model with C—H distances of 0.93Å (*C*-aromatic) and 0.96Å (*C*-methyl).

All H atoms were refined with isotropic displacement parameters were set at 1.2 *U*_eq_(C-aromatic, N) and 1.5 *U*_eq_(*C*-methyl).

The U^ij^ components of O4 were restrained to approximate isotropic behaviour.

### Crystal data

**Table:**

C_14_H_12_N_2_O_5_S	*F*(000) = 1328
*M**_r_* = 320.32	*D*_x_ = 1.500 Mg m^−^^3^
Orthorhombic, *P**b**c**a*	Mo *K*α radiation, λ = 0.71073 Å
Hall symbol: -P 2ac 2ab	Cell parameters from 818 reflections
*a* = 13.969 (1) Å	θ = 2.6–27.9°
*b* = 9.6591 (6) Å	µ = 0.25 mm^−^^1^
*c* = 21.026 (2) Å	*T* = 293 K
*V* = 2837.0 (4) Å^3^	Rod, colourless
*Z* = 8	0.40 × 0.18 × 0.18 mm

### Data collection

**Table:**

Oxford Diffraction Xcalibur diffractometer with a Sapphire CCD detector	2864 independent reflections
Radiation source: fine-focus sealed tube	1836 reflections with *I* > 2σ(*I*)
Graphite monochromator	*R*_int_ = 0.027
ω and φ scans	θ_max_ = 26.4°, θ_min_ = 2.9°
Absorption correction: multi-scan (*CrysAlis RED*; Oxford Diffraction, 2009)	*h* = −7→17
*T*_min_ = 0.905, *T*_max_ = 0.956	*k* = −7→12
7163 measured reflections	*l* = −26→19

### Refinement

**Table:**

Refinement on *F*^2^	Primary atom site location: structure-invariant direct methods
Least-squares matrix: full	Secondary atom site location: difference Fourier map
*R*[*F*^2^ > 2σ(*F*^2^)] = 0.062	Hydrogen site location: inferred from neighbouring sites
*wR*(*F*^2^) = 0.152	H atoms treated by a mixture of independent and constrained refinement
*S* = 1.16	*w* = 1/[σ^2^(*F*_o_^2^) + (0.0409*P*)^2^ + 3.5546*P*] where *P* = (*F*_o_^2^ + 2*F*_c_^2^)/3
2863 reflections	(Δ/σ)_max_ = 0.015
203 parameters	Δρ_max_ = 0.32 e Å^−^^3^
7 restraints	Δρ_min_ = −0.21 e Å^−^^3^

### Special details

**Table:**

Geometry. All s.u.'s (except the s.u. in the dihedral angle between two l.s. planes) are estimated using the full covariance matrix. The cell s.u.'s are taken into account individually in the estimation of s.u.'s in distances, angles and torsion angles; correlations between s.u.'s in cell parameters are only used when they are defined by crystal symmetry. An approximate (isotropic) treatment of cell s.u.'s is used for estimating s.u.'s involving l.s. planes.
Refinement. Refinement of *F*^2^ against ALL reflections. The weighted *R*-factor *wR* and goodness of fit *S* are based on *F*^2^, conventional *R*-factors *R* are based on *F*, with *F* set to zero for negative *F*^2^. The threshold expression of *F*^2^ > σ(*F*^2^) is used only for calculating *R*-factors(gt) *etc*. and is not relevant to the choice of reflections for refinement. *R*-factors based on *F*^2^ are statistically about twice as large as those based on *F*, and *R*- factors based on ALL data will be even larger.

### Fractional atomic coordinates and isotropic or equivalent isotropic displacement parameters (Å^2^)

**Table:**

	*x*	*y*	*z*	*U*_iso_*/*U*_eq_	
C1	0.5261 (3)	0.2260 (4)	0.55537 (17)	0.0407 (9)	
C2	0.4949 (3)	0.1161 (4)	0.5903 (2)	0.0490 (10)	
H2	0.5302	0.0345	0.5911	0.059*	
C3	0.4113 (3)	0.1260 (4)	0.6240 (2)	0.0533 (11)	
H3	0.3899	0.0521	0.6486	0.064*	
C4	0.3601 (3)	0.2459 (4)	0.62105 (19)	0.0450 (9)	
C5	0.3893 (3)	0.3565 (5)	0.5862 (2)	0.0669 (13)	
H5	0.3531	0.4372	0.5847	0.080*	
C6	0.4737 (4)	0.3461 (5)	0.5534 (2)	0.0708 (14)	
H6	0.4956	0.4209	0.5296	0.085*	
C7	0.7385 (3)	0.3573 (4)	0.59457 (17)	0.0405 (9)	
C8	0.8213 (3)	0.3558 (4)	0.63877 (17)	0.0385 (8)	
C9	0.9003 (3)	0.2694 (4)	0.63104 (19)	0.0449 (9)	
H9	0.9018	0.2066	0.5975	0.054*	
C10	0.9763 (3)	0.2765 (4)	0.67281 (19)	0.0490 (10)	
H10	1.0293	0.2199	0.6664	0.059*	
C11	0.9750 (3)	0.3668 (4)	0.72441 (19)	0.0463 (10)	
C12	0.8959 (3)	0.4520 (4)	0.73124 (19)	0.0497 (10)	
H12	0.8938	0.5135	0.7652	0.060*	
C13	0.8207 (3)	0.4481 (4)	0.68931 (18)	0.0458 (10)	
H13	0.7691	0.5076	0.6948	0.055*	
C14	1.0575 (3)	0.3736 (5)	0.7701 (2)	0.0638 (12)	
H14A	1.1160	0.3865	0.7469	0.077*	
H14B	1.0609	0.2889	0.7939	0.077*	
H14C	1.0484	0.4499	0.7988	0.077*	
N1	0.7201 (2)	0.2339 (3)	0.56360 (15)	0.0421 (8)	
H1N	0.750 (3)	0.161 (3)	0.5748 (17)	0.051*	
N2	0.2706 (3)	0.2572 (4)	0.65756 (17)	0.0569 (9)	
O1	0.6446 (2)	0.0697 (3)	0.49280 (14)	0.0605 (8)	
O2	0.6361 (2)	0.3183 (3)	0.46528 (13)	0.0647 (9)	
O3	0.68781 (18)	0.4583 (3)	0.58609 (13)	0.0519 (7)	
O4	0.2492 (3)	0.1639 (4)	0.6926 (2)	0.1066 (14)	
O5	0.2239 (2)	0.3613 (4)	0.65262 (17)	0.0805 (10)	
S1	0.63333 (7)	0.21032 (11)	0.51121 (5)	0.0474 (3)	

### Atomic displacement parameters (Å^2^)

**Table:**

	*U*^11^	*U*^22^	*U*^33^	*U*^12^	*U*^13^	*U*^23^
C1	0.042 (2)	0.042 (2)	0.038 (2)	−0.0042 (18)	−0.0084 (17)	0.0030 (17)
C2	0.046 (2)	0.035 (2)	0.067 (3)	0.0027 (18)	−0.002 (2)	0.005 (2)
C3	0.050 (2)	0.039 (2)	0.070 (3)	−0.003 (2)	0.003 (2)	0.012 (2)
C4	0.0384 (19)	0.044 (2)	0.053 (2)	−0.0040 (18)	−0.0034 (19)	0.0001 (18)
C5	0.061 (3)	0.052 (3)	0.088 (4)	0.018 (2)	0.014 (3)	0.024 (3)
C6	0.073 (3)	0.053 (3)	0.087 (3)	0.012 (2)	0.019 (3)	0.039 (3)
C7	0.041 (2)	0.0330 (19)	0.048 (2)	−0.0044 (17)	0.0068 (18)	−0.0038 (18)
C8	0.0351 (18)	0.0321 (18)	0.048 (2)	−0.0054 (16)	0.0051 (17)	−0.0010 (17)
C9	0.046 (2)	0.035 (2)	0.054 (2)	−0.0011 (18)	0.0016 (19)	−0.0090 (18)
C10	0.041 (2)	0.042 (2)	0.064 (3)	0.0021 (18)	0.002 (2)	−0.001 (2)
C11	0.043 (2)	0.049 (2)	0.047 (2)	−0.007 (2)	0.0037 (18)	0.001 (2)
C12	0.048 (2)	0.056 (2)	0.045 (2)	−0.007 (2)	0.0065 (19)	−0.013 (2)
C13	0.040 (2)	0.044 (2)	0.053 (2)	0.0000 (18)	0.0099 (19)	−0.0075 (19)
C14	0.055 (3)	0.085 (3)	0.052 (3)	−0.005 (2)	−0.007 (2)	−0.002 (2)
N1	0.0432 (18)	0.0345 (17)	0.0487 (19)	−0.0012 (14)	−0.0045 (15)	−0.0009 (15)
N2	0.051 (2)	0.056 (2)	0.064 (2)	−0.0004 (19)	0.0042 (19)	0.0004 (19)
O1	0.0604 (18)	0.0604 (18)	0.0606 (18)	−0.0056 (15)	0.0001 (15)	−0.0237 (15)
O2	0.069 (2)	0.081 (2)	0.0444 (16)	−0.0037 (17)	0.0040 (15)	0.0182 (16)
O3	0.0455 (16)	0.0353 (14)	0.0749 (19)	0.0039 (13)	−0.0010 (14)	−0.0027 (14)
O4	0.098 (3)	0.081 (2)	0.140 (3)	0.004 (2)	0.058 (2)	0.026 (2)
O5	0.062 (2)	0.086 (2)	0.094 (3)	0.0287 (19)	0.0031 (18)	0.004 (2)
S1	0.0490 (6)	0.0523 (6)	0.0409 (5)	−0.0053 (5)	−0.0008 (5)	−0.0027 (5)

### Geometric parameters (Å, º)

**Table:**

C1—C2	1.362 (5)	C9—H9	0.9300
C1—C6	1.373 (5)	C10—C11	1.392 (5)
C1—S1	1.769 (4)	C10—H10	0.9300
C2—C3	1.369 (5)	C11—C12	1.385 (5)
C2—H2	0.9300	C11—C14	1.502 (5)
C3—C4	1.363 (5)	C12—C13	1.372 (5)
C3—H3	0.9300	C12—H12	0.9300
C4—C5	1.359 (6)	C13—H13	0.9300
C4—N2	1.471 (5)	C14—H14A	0.9600
C5—C6	1.369 (6)	C14—H14B	0.9600
C5—H5	0.9300	C14—H14C	0.9600
C6—H6	0.9300	N1—S1	1.654 (3)
C7—O3	1.218 (4)	N1—H1N	0.852 (19)
C7—N1	1.383 (4)	N2—O4	1.202 (5)
C7—C8	1.484 (5)	N2—O5	1.203 (4)
C8—C13	1.387 (5)	O1—S1	1.421 (3)
C8—C9	1.393 (5)	O2—S1	1.422 (3)
C9—C10	1.380 (5)		
			
C2—C1—C6	120.3 (4)	C11—C10—H10	119.4
C2—C1—S1	119.1 (3)	C12—C11—C10	117.6 (4)
C6—C1—S1	120.5 (3)	C12—C11—C14	121.3 (4)
C1—C2—C3	119.9 (4)	C10—C11—C14	121.1 (4)
C1—C2—H2	120.1	C13—C12—C11	121.9 (4)
C3—C2—H2	120.1	C13—C12—H12	119.1
C4—C3—C2	118.9 (4)	C11—C12—H12	119.1
C4—C3—H3	120.6	C12—C13—C8	120.4 (4)
C2—C3—H3	120.6	C12—C13—H13	119.8
C5—C4—C3	122.4 (4)	C8—C13—H13	119.8
C5—C4—N2	118.6 (4)	C11—C14—H14A	109.5
C3—C4—N2	119.0 (4)	C11—C14—H14B	109.5
C4—C5—C6	118.2 (4)	H14A—C14—H14B	109.5
C4—C5—H5	120.9	C11—C14—H14C	109.5
C6—C5—H5	120.9	H14A—C14—H14C	109.5
C5—C6—C1	120.4 (4)	H14B—C14—H14C	109.5
C5—C6—H6	119.8	C7—N1—S1	124.7 (3)
C1—C6—H6	119.8	C7—N1—H1N	119 (3)
O3—C7—N1	120.9 (3)	S1—N1—H1N	116 (3)
O3—C7—C8	123.5 (3)	O4—N2—O5	123.0 (4)
N1—C7—C8	115.6 (3)	O4—N2—C4	118.4 (4)
C13—C8—C9	118.6 (4)	O5—N2—C4	118.6 (4)
C13—C8—C7	118.0 (3)	O1—S1—O2	120.89 (19)
C9—C8—C7	123.4 (3)	O1—S1—N1	103.42 (17)
C10—C9—C8	120.4 (4)	O2—S1—N1	109.36 (17)
C10—C9—H9	119.8	O1—S1—C1	108.56 (18)
C8—C9—H9	119.8	O2—S1—C1	108.45 (18)
C9—C10—C11	121.2 (4)	N1—S1—C1	105.02 (16)
C9—C10—H10	119.4		
			
C6—C1—C2—C3	−0.7 (6)	C14—C11—C12—C13	179.1 (4)
S1—C1—C2—C3	−179.1 (3)	C11—C12—C13—C8	1.0 (6)
C1—C2—C3—C4	1.1 (6)	C9—C8—C13—C12	−1.0 (5)
C2—C3—C4—C5	−0.6 (7)	C7—C8—C13—C12	−179.4 (3)
C2—C3—C4—N2	−179.6 (4)	O3—C7—N1—S1	2.5 (5)
C3—C4—C5—C6	−0.3 (7)	C8—C7—N1—S1	−178.8 (3)
N2—C4—C5—C6	178.7 (4)	C5—C4—N2—O4	−173.7 (5)
C4—C5—C6—C1	0.7 (8)	C3—C4—N2—O4	5.4 (6)
C2—C1—C6—C5	−0.2 (7)	C5—C4—N2—O5	3.9 (6)
S1—C1—C6—C5	178.2 (4)	C3—C4—N2—O5	−177.0 (4)
O3—C7—C8—C13	25.4 (5)	C7—N1—S1—O1	179.3 (3)
N1—C7—C8—C13	−153.2 (3)	C7—N1—S1—O2	49.2 (4)
O3—C7—C8—C9	−152.8 (4)	C7—N1—S1—C1	−67.0 (3)
N1—C7—C8—C9	28.5 (5)	C2—C1—S1—O1	29.9 (4)
C13—C8—C9—C10	−0.3 (6)	C6—C1—S1—O1	−148.6 (4)
C7—C8—C9—C10	177.9 (3)	C2—C1—S1—O2	162.9 (3)
C8—C9—C10—C11	1.8 (6)	C6—C1—S1—O2	−15.5 (4)
C9—C10—C11—C12	−1.8 (6)	C2—C1—S1—N1	−80.2 (3)
C9—C10—C11—C14	179.6 (4)	C6—C1—S1—N1	101.3 (4)
C10—C11—C12—C13	0.4 (6)		

### Hydrogen-bond geometry (Å, º)

**Table:**

*D*—H···*A*	*D*—H	H···*A*	*D*···*A*	*D*—H···*A*
N1—H1*N*···O3^i^	0.85 (2)	2.16 (2)	2.994 (4)	168 (4)

Symmetry code: (i) −*x*+3/2, *y*−1/2, *z*.
